# Functional Recovery Levels, Associated Clinical Features and the Role of Metabolic Syndrome in Schizophrenia Patients Followed in a University Hospital

**DOI:** 10.1192/j.eurpsy.2025.566

**Published:** 2025-08-26

**Authors:** G. Şimşek Avcı

**Affiliations:** psychiatry, Gazi university hospital, ankara, Türkiye

## Abstract

**Introduction:**

Schizophrenia is a chronic illness that causes severe disability and dysfunction.The traditional approach focusing on symptom control does not always result in improvement in functioning. Functional recovery is considered to be the achievement of social and occupational functioning and independent living in addition to symptom remission.Factors like negative symptoms, depression, cognitive dysfunction, treatment compliance, internalised stigma, and education impact functional recovery. MetS may impact functional recovery by contributing to depression, reducing treatment compliance, and impairing cognitive functions, but studies on this are limited.

**Objectives:**

This study aimed to investigate the relationship between MetS and functional recovery in schizophrenia, along with related clinical features.

**Methods:**

The study sample included 115 schizophrenia patients aged 18-65, who applied to Gazi University Psychiatry Outpatient Clinic, spoke Turkish, no exacerbation in the last year.Exclusion criteria were serious medical/neurological illness, alcohol/substance use disorder.MetS was diagnosed per American College of Cardiology criteria. Functional Remission of General Schzophrenia Scale (FROGS), Schizophrenia Cognition Rating Scale (SCoRS), Positive and Negative Syndrome Scale (PANSS), The Calgary Depression Scale for Schizophrenia (CDSS), Medication Adherence Rating Scale (MARS), Schedule for Assesing the Three Components of Insight (SAI), Internalized Stigma of Mental Illness Scale (ISMI) scales were applied to all participants. SPSS 22.0 was used, and p<0.05 was considered significant.

**Results:**

The mean age of participants was 48.61, 54.8% were male and 44% had a high school education. MetS was 55.7% of patients.Patients with MetS had significantly lower scores of FROGS, showed more cognitive impairment in problem solving, there were no significant differences between the groups in the other variables examined.The proportion of patients with MetS (79.7%) showing low level of functionality was higher than those without MetS.The total score of FROGS was positively correlated with years of education, scores of MARS and SAI, while it was negatively correlated with age and CDSS scores.Among the components of MetS, fasting glucose level and diastolic blood pressure were found to be significantly correlated with the scores of the FROGS.The negative predictors of functioning were found to be education level, MARS scores, MetS, SCoRS attention domain and PANSS negative scores.

**Conclusions:**

Our results show that MetS associated with lower functionality in schizophrenia. Therefore, good metabolic control in patients with schizophrenia is important for cognitive skills and functionality as well as physical health.

**Disclosure of Interest:**

None Declared

